# Buprenorphine reverses neurocognitive impairment in EcoHIV infected mice: A potential therapy for HIV-NCI

**DOI:** 10.3389/fimmu.2022.1004985

**Published:** 2022-10-07

**Authors:** Aniella J. Murphy, Jennifer Kelschenbach, Hongxia He, Wei Chao, Boe-Hyun Kim, David J. Volsky, Joan W. Berman

**Affiliations:** ^1^ Laboratory of Dr. Joan W. Berman, Department of Pathology, Albert Einstein College of Medicine, Bronx, NY, United States; ^2^ Laboratory or Dr. David J. Volsky, Department of Medicine, Icahn School of Medicine at Mount Sinai, Manhattan, NY, United States

**Keywords:** neuropathogenesis, HIV, monocytes, buprenorphine, cognitive impairment

## Abstract

Thirty-eight million people worldwide are living with HIV, PWH, a major public health problem. Antiretroviral therapy (ART) revolutionized HIV treatment and significantly increased the lifespan of PWH. However, approximately 15-50% of PWH develop HIV associated neurocognitive disorders (HIV-NCI), a spectrum of cognitive deficits, that negatively impact quality of life. Many PWH also have opioid use disorder (OUD), and studies in animal models of HIV infection as well as in PWH suggest that OUD can contribute to HIV-NCI. The synthetic opioid agonist, buprenorphine, treats OUD but its effects on HIV-NCI are unclear. We reported that human mature inflammatory monocytes express the opioid receptors MOR and KOR, and that buprenorphine reduces important steps in monocyte transmigration. Monocytes also serve as HIV reservoirs despite effective ART, enter the brain, and contribute to HIV brain disease. Using EcoHIV infected mice, an established model of HIV infection and HIV-NCI, we previously showed that pretreatment of mice prior to EcoHIV infection reduces mouse monocyte entry into the brain and prevents NCI. Here we show that buprenorphine treatment of EcoHIV infected mice with already established chronic NCI completely reverses the disease. Disease reversal was associated with a significant reduction in brain inflammatory monocytes and reversal of dendritic injury in the cortex and hippocampus. These results suggest that HIV-NCI persistence may require a continuing influx of inflammatory monocytes into the brain. Thus, we recommend buprenorphine as a potential therapy for mitigation of HIV brain disease in PWH with or without OUD.

## Introduction

Thirty eight million people globally, including 1.2 million in the US, are living with HIV, PWH (1). Antiretroviral therapy (ART) revolutionized HIV treatment, enabling long-term suppression of HIV replication, improvement in immune functions, and increased lifespan (2). Despite the success of ART, 15-50% of PWH develop HIV associated neurocognitive impairment, HIV-NCI (3–8). HIV-NCI is a spectrum of cognitive deficits that negatively impact the quality of life for PWH and is an independent risk factor for morbidity and mortality (5, 6, 9, 10). Despite several successful treatment attempts for HIV-NCI in preclinical animal models no approved therapy for NCI in PWH has emerged (11–17).

HIV enters the brain early after peripheral infection, establishing a long-lived viral reservoir in myeloid cells in the CNS that is difficult to detect and is not eliminated by ART (6, 18, 19). This reservoir mediates ongoing low level neuroinflammation and CNS damage (20–23). One major proposed mechanism responsible for the establishment and reseeding of the HIV CNS reservoir and associated neuroinflammation is the migration of HIV infected and uninfected CD14+ CD16+ (mature) monocytes into the brain (22, 24–28). These cells can serve as a source of neurotoxic host and viral products and contribute to cognitive dysfunction (23, 29–32). Studies have shown that mature monocytes cross the blood brain barrier, BBB, and enter the brain in response to chemokines including CCL2 (18, 33–36). These cells can also differentiate into long lived perivascular macrophages (37). Increased presence of perivascular CD163-positive macrophages in the brain correlates with CNS disease progression in SIV infected non-human primates (38). EcoHIV infected athymic mice were shown to have increased numbers of inflammatory macrophages in the brain and develop HIV-NCI-like disease despite a lack of mature T lymphocytes, suggesting that macrophage infection is sufficient for causing HIV neuropathogenesis (39). Similarly, HIV infection of mice reconstituted with human myeloid precursor cells results in pathologic and virologic markers of HIV brain disease (40). Other studies in non-human primates and mice have also underscored the importance of monocytes in this process (41–44). There are also data that show a correlation between HIV DNA in mature monocytes and cognitive impairment in PWH (27, 28, 45). Thus, monocyte migration to the CNS may be a critical step in HIV-NCI neuropathogenesis to target for future therapies.

Opioid use disorder, OUD, significantly impacts the quality of life for PWH, reduces ART adherence, and is associated with worse cognitive outcomes in some individuals (46–49). Buprenorphine, an opioid derivative, is a commonly used opioid agonist therapy (OAT) to treat OUD (50–53). In some studies, buprenorphine was shown to improve neuropsychological outcomes in people with OUD, as well as in a small study including PWH with OUD (54–56). The effects of buprenorphine on mechanisms of HIV neuropathogenesis remain unclear. Buprenorphine is a partial agonist of the mu opioid receptor, MOR, and a full antagonist of the kappa opioid receptor, KOR (52). These receptors are expressed on mature monocytes, macrophages, and cells of the CNS indicating that buprenorphine can bind to these cells and modulate their functions (57–59). We previously showed that buprenorphine decreases critical steps in human monocyte transmigration across the BBB, including CCL2 mediated adhesion and chemotaxis (57, 60). These findings suggest that buprenorphine may improve neurocognitive outcomes in PWH by limiting the entry of HIV infected and uninfected monocytes into the brain.

In our previous study, we used buprenorphine as a prophylactic treatment three days before and during EcoHIV infection of immunocompetent mice, an established animal model of naturally suppressed HIV infection and HIV-NCI-like disease, EcoHIV-NCI (61). When daily buprenorphine injections were initiated prior to EcoHIV infection and continued for four weeks until evaluation, EcoHIV-NCI was prevented (61). This was correlated with a marked reduction of inflammatory monocytes in the brain and no effect on peripheral infection (61). These results showed that buprenorphine prophylaxis does not affect systemic EcoHIV infection and suggested that limiting monocyte transmigration into the CNS prevents processes required for HIV-NCI manifestation in mice.

In this present study, we examined whether ongoing entry of inflammatory monocytes into the brain is required after persistent NCI has been established and whether buprenorphine can interrupt this process and potentially reverse cognitive impairment. We show that daily buprenorphine treatment of cognitively impaired mice reverses the disease along with significant reductions in brain inflammatory monocytes, HIV DNA in the brain, and hippocampal and cortical synaptodendritic injury. These results indicate that HIV-NCI persistence may require a continued influx of inflammatory monocytes and that buprenorphine could be a potential therapy for HIV CNS disease in PWH with or without OUD.

## Materials and methods

### Mice

Eight-week-old, male, C57BL/6J (Jackson Laboratory, Bar Harbor, ME) were used. We maintained the mice under appropriate husbandry conditions and ensured all animals had minimized distress, discomfort, and injury. All studies were approved by the Icahn School of Medicine at Mount Sinai and the Albert Einstein College of Medicine Institutional Animal Care and Use Committees and are in compliance with the US Animal Welfare Act PHS policies, animal welfare assurance D16-00069.

### Buprenorphine injections, EcoHIV infection, and tissue collection

Four treatment groups of mice were used: Control (CTRL), did not receive buprenorphine or virus; ECOHIV, was infected but did not receive buprenorphine; ECOHIV BUP, was infected and received buprenorphine; and BUP, received buprenorphine and was not infected. Mice were infected with a single intraperitoneal dose of 2 x 10^6^ pg of EcoHIV p24 or with diluent control, PBS. Buprenorphine was injected subcutaneously, daily, at a dose of 0.2 mg/kg (NIDA, Rockville, MA), which was begun at either two weeks or four weeks post-infection. This represents an intermediate maintenance dose in humans, based upon published findings (62). For our studies we used two different treatment timepoints. In the first timepoint, daily buprenorphine injections were given after two weeks of EcoHIV infection, which is an earlier timepoint at which NCI manifests, and continued daily for an additional two weeks. After a total of four weeks, mice underwent behavioral testing. Daily injections of buprenorphine were given throughout behavioral testing until time of sacrifice. For the second timepoint daily buprenorphine injections were started four weeks after EcoHIV infection and continued daily for an additional two weeks. After a total of six weeks, mice underwent behavioral testing, with daily buprenorphine injections until sacrifice. This strategy demonstrates the ability of buprenorphine to treat NCI when given after chronic infection. The untreated groups were injected daily with vehicle (sterile water) to ensure that all mice were handled similarly. At the end of the experiment mice were anesthetized with ketamine (100 mg/kg)/xylazine (5mg/kg), intracardiac perfused with cold HBSS, and spleens and brain tissues were removed and prepared for measurement of monocytes, HIV burden, and microscopy as described (13, 39, 61, 63).

### Radial arm water maze

The radial arm water maze (RAWM) test was administered in a pool of opaque water containing six swimming lanes and a hidden platform as described (13, 39, 63). Briefly, each testing group contained eight mice. RAWM testing consisted of four training trials (T) of 60s and one post-training 60s retention trial (RT) administered after 30 min rest. The hidden platform was rotated randomly to a different arm each test day to ensure that mice used working memory to locate the platform. Testing was considered complete when control mice reached asymptotic performance of one error or fewer in finding the hidden platform on trials T4 and RT. We recorded two measures of cognitive performance, errors and latency. An error is defined as the animal swimming into an arm that does not contain the hidden platform or remaining inactive for 20s, and latency is the amount of time it takes the mouse to find the hidden platform. Errors and latency for the last three days of testing were averaged and used for statistical analysis.

### Isolation of CNS immune cells

The brains from seven of the mice per group that underwent water maze testing were removed and brain immune cells isolated using a previously published protocol (61). In brief, a cell suspension was generated from isolated brain tissue using a 100uM cell filter. The cell suspension was treated with Liberase TL (Roche Diagnostics) and 50 uL of DNAse1 (1 mg/ml, Stem Cell, technologies) to digest connective tissue. The digested suspension was filtered through a 70uM filter, and cells were isolated using Percoll density centrifugation. Cells obtained from this isolation method contain the migratory and nonmigratory cells in the brain. We identified and quantified monocytes from individual mouse brains by flow cytometry using a staining and gating strategy for CD45 (hematopoietic cells, Biolegend, 1ug), CD11b (myeloid cells, Biolegend, 0.25ug), Ly6G (granulocytes, BD, 0.25ug), and Ly6C (inflammatory monocytes, Biolegend, 0.25ug). Fluorescence minus one (FMO) controls were used to establish appropriate cell gates. Samples were acquired using an Attune NxT flow cytometer (Invitrogen, Carlsbad, CA, USA) and analyzed with FlowJo version 10.8.0.

### Detection of peripheral HIV DNA

For all experiments spleen cells were harvested and DNA isolated from four to five mice in each treatment group. EcoHIV infection was quantified by real-time quantitative PCR (QPCR) for EcoHIV *gag* and compared between ECOHIV and ECOHIV BUP groups. The procedures for harvesting brain tissues or spleen, preparation of cellular DNA, and detection of EcoHIV *gag* QPCR were described previously (13, 39, 57, 64–66). Samples for QPCR were run in duplicate in an QuantStudio™ 3 Real-Time PCR System (Thermo Fisher Scientific). DNA QPCR reactions were normalized by amplification of glyceraldehyde 3-phosphate dehydrogenase (GAPDH) using a kit from Applied Biosystems.

### Detection of brain HIV DNA

For QPCR, DNA was isolated from brain tissues by using a modified TRIzol (Invitrogen) protocol and an auto-homogenizer (Next Advance Inc., Bullet Blender Storm 24) (67). Expression of EcoHIV *gag* was determined as described above. Samples for QPCR were run in duplicate in an QuantStudio™ 3 Real-Time PCR System. DNA QPCR reactions were normalized to GAPDH and presented as absolute expression levels. For droplet digital PCR studies (ddPCR), DNA was isolated from an anterior coronal portion of the right hemisphere from four to five mice in each treatment group according to manufacturer’s instructions (Qiagen co isolation of DNA/RNA kit). After isolation, DNA from each brain per group was pooled and concentrated (Zymo DNA concentrator kit). Analysis of HIV *gag* DNA copies was performed using a Bio-Rad QX-100 system (BioRad) and a custom primer and probe set for HIV *gag* (HIV *gag* F: 5-TGGGACCACAGGCTACACTAGA-3, R: 5-CAGCCAAAACTCTTGCTTTATGG-3, P: 5-TGATGACAGCATGCCAGGGAGT-3). The number of cells in each sample was determined using a GAPDH qPCR standard curve. HIV *gag* DNA copies were determined by ddPCR and normalized to 1 million cells.

### Immunofluorescent staining and confocal microscopy

Mice (3/group) were perfused with 4% paraformaldehyde (PFA) followed by saline. Following perfusion, the brain from each mouse was removed and placed in 4% PFA for preservation and kept at 4 degrees until sectioning. Prior to sectioning, brains were re-hydrated using a gradient of glucose and then the entire brain was frozen and then embedded in optimal cutting temperature (OCT) compound. The entire brain was cut using a cryostat (Leica) into 30 uM sections. Ten to 20 floating sections per mouse which contained the area of interest (hippocampus) were mounted onto slides for staining. Sections were stained with the following antibodies: rabbit anti-microtubule-associated protein 2 (MAP2) for detection of dendrites (1:150, EMD Millipore, Mahopac, NY) and mouse monoclonal anti-neuronal nuclear antigen (NeuN) for detection of neuronal nuclei (1:150, EMD Millipore), followed by matching Alexa conjugated secondary antibodies (1:100, Thermo Fisher Scientific). Images were captured by two investigators blinded to the treatment groups with a motorized Leica TCS SP5 confocal microscope, and analyzed using Improvision Volocity software (PerkinElmer) as described (13, 65, 66). Quantification of images was performed using ten-20 images (2-4 sections/mouse, 3 mice/group) measuring intensity of staining for MAP2 in the CA3 region of the hippocampus and in the cortex dorsal to the hippocampus.

### Statistical analysis

One-way ANOVA tests were performed to compare differences in errors, latency, and monocyte numbers between each group. P-values of statistical comparisons between CTRL and ECOHIV groups are represented by asterisks (*P < 0.05, **P < 0.05, ***P < 0.0005, ****P < 0.001), and differences between CTRL and ECOHIV BUP groups are represented by ampersands (^&^P < 0.05, ^&&^P < 0.005, ^&&&^P < 0.0005, and ^&&&&^P < 0.0001). A student t-test or the nonparametric Mann-Whitney, non parametric, t-test was used to compare the differences in EcoHIV peripheral and brain viral burden between ECOHIV and ECOHIV BUP groups. Watermaze and qPCR are shown as mean +/- SEM, and monocyte data are shown as mean +/- SD. Staining intensities are shown as mean +/- SEM and significance was calculated using a two-tailed Student’s t-test. All analyses were performed on GraphPad Prism version 9.2.0.

## Results

### Buprenorphine reverses EcoHIV NCI, reduces monocyte migration into the CNS, and viral brain DNA when given two weeks after infection

The transmigration of inflammatory monocytes into the brain is an important mechanism of HIV neuropathogenesis (19, 26, 27, 42). It has been shown that mature monocytes from PWH transmigrate in greater numbers across the BBB compared to those from people who do not have HIV. We showed that chronically EcoHIV infected nude (39) or wild type mice (61) with NCI also have elevated levels of inflammatory macrophages/monocytes in the brain. One way to mitigate HIV neuropathogenesis may be by reducing the influx of inflammatory monocytes into the brain. Therefore, we studied whether buprenorphine could treat and reverse cognitive impairment when given after established EcoHIV infection, and whether it would also reduce the number of brain inflammatory monocytes and the amount of HIV DNA in the brain.

**Figure 1:**
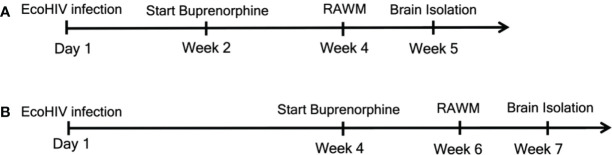
Experimental outline for the two treatment timepoints. We began daily buprenorphine injections at two different timepoints. For **(A)** mice were infected with EcoHIV at Day 1. Buprenorphine injections started two weeks later and were administered daily for the remainder of the experiments. After the additional two weeks, for a total of four weeks, mice underwent RAWM testing to assess cognitive status. RAWM testing was for one week, after which brains were isolated for monocytes, HIV DNA, and synaptodendritic pruning studies. For **(B)**, mice were infected and daily buprenorphine injections began four weeks later and continued for the remainder of the experiments. At week six, mice were assessed for cognitive impairment using the RAWM, and at week seven brains were isolated for monocyte and virological assessment.

**Figure 2:**
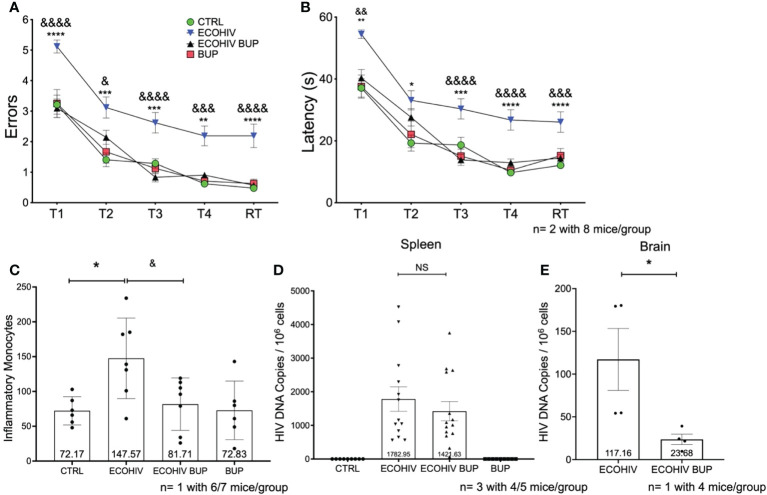
Buprenorphine reverses cognitive impairment, decreases inflammatory monocytes and HIV DNA in the brain when given two weeks post-EcoHIV infection. C57BL/6J mice in each experimental group; CTRL (green circles), ECOHIV (blue inverted triangles), ECOHIV BUP (black triangles), and BUP (red squares) were tested using a radial arm water maze. Mice were infected for two weeks before buprenorphine injections began. After an additional two weeks, mice were assayed for cognitive performance using the RAWM **(A, B)**. EcoHIV infected mice were cognitively impaired after four weeks of infection as these mice made significantly more errors **(A)** and took significantly longer **(B)** to find the hidden platform compared to both CTRL (*) and ECOHIV BUP (&) groups. Data are shown as mean+/- SEM from the last three days of water maze testing. There were a total of two independent experiments, each with eight mice/group for a total of 16 mice in the CTRL, ECOHIV, and ECOHIV BUP groups. One independent experiments was performed with 8 mice for the BUP group **(A, B)** ECOHIV vs CTRL, **p<0.005, ****p<0.0001. ECOHIV vs ECOHIV BUP, ^&^p<0.05, ^&&^p<0.005, ^&&&^p<0.0005, ^&&&&^p<0.0001. **(C)**, Immune cells from the brains of six to seven mice in each group in one independent experiment that underwent water maze testing were isolated and the number of inflammatory monocytes quantified by flow cytometry. The data are shown as mean +/- SD and analyzed using a one way ANOVA. There is a significant increase (147.57 +/- 57.83) in the number of inflammatory monocytes in the brains of EcoHIV infected mice compared to control (72.17 +/- 20.27, *p<0.05). These data also show that buprenorphine treatment given two weeks post infection significantly reduces the number of brain inflammatory monocytes (81.71 +/- 37.65, ^&^p<0.05). **(D)**, Spleens were taken and HIV *gag* DNA quantified from four or five mice in each group in three independent experiments. These data show that buprreonorphine does not change peripheral infection. **(E)**, DNA was isolated from brain tissue of four mice in each group in one independent experiment. Using qPCR we demonstrated that buprenorphine treatments, started after two weeks, significantly decreases the amount of HIV DNA in the brain of infected mice (23.68 +/- 12.12, *p<0.05); ***p<0.0005; NS, Not Significant.

We initiated buprenorphine treatment two or four weeks after EcoHIV infection of mice (**Figure 1**). The results of the two week post infection treatment testing for HIV-NCI, inflammatory monocyte transmigration to brain, and HIV burdens are shown in **Figure 2**. Buprenorphine or vehicle were administered daily starting two week post infection for a total of three weeks including the time required for completion of the five day RAWM test (**Figure 1** **)**. Male C57BL/6J mice (n=10-15) were infected with a single intraperitoneal dose of EcoHIV or with PBS as described in the methods. There were four experimental groups: CTRL, ECOHIV, ECOHIV BUP, and BUP. Eight mice from each group in two independent experiments, for a total of 16 mice, underwent water maze testing using our established protocol. We assessed two measures of cognitive performance, errors and latency. Errors are defined as a mouse swimming into an arm that does not contain the hidden platform or remaining inactive for 20s. Latency is the amount of time the mouse takes to find the platform. RAWM results were analyzed using a one way ANOVA. EcoHIV infected mice were cognitively impaired as indicated by a significant increase in the number of errors made (T1: p<0.0001, T2-T3: p<0.0005, T4: p<0.005, RT: p<0.0001) and latency to find (T1: p<0.005, T2: p<0.05, T3: p<0.0005, T4-RT: p<0.0001) the hidden platform. In contrast, ECOHIV BUP mice were not impaired as they made significantly fewer errors (T1: p<0.0001, T2: p<0.05, T3: p<0.0001, T4: p<0.0005, RT: p<0.0001) and took less time (T1: p<0.005, T3-T4: p<0.0001, RT: p<0.0005) to find the platform compared to ECOHIV (**Figures 2A, B**, ECOHIV BUP versus ECOHIV). In fact, ECOHIV BUP mice behaved similarly to CTRL, with no significant differences in either errors or latency in water maze testing between the two groups. In this study and others, we also confirmed that buprenorphine treatments did not alter the time to find a visible platform, which serves as a control test to assess whether treatments and conditions tested result in any changes in motivation or vision. These results show that buprenorphine can treat and reverse EcoHIV-NCI in this animal model when treatment is begun two weeks after infection.

Next, we determined the effect of buprenorphine treatment on the migration of inflammatory monocytes into the brain (**Figure 2C**) and virus burdens in spleen cells and brains (**Figures 2D, E**). Immune cells were extracted from six or seven perfused brains of mice that completed cognitive testing and monocytes were quantified using a previously established protocol (61). This gating strategy for identifying inflammatory brain monocytes is shown in **Supplementary Figure 1**. EcoHIV infected mice had a significantly increased number of brain inflammatory monocytes (147.57 +/- 57.83) compared to control mice (72.17 +/- 20.27, p < 0.05). In contrast, the number of these cells in buprenorphine treated, EcoHIV infected mice was significantly lower than in infected mice (81.71 +/- 37.65, p < 0.05 ECOHIV BUP vs ECOHIV) and did not differ from control mice (**Figure 2C**). Buprenorphine alone had no effect on brain inflammatory monocyte levels compared to control mice. Thus, buprenorphine treatment started two weeks after EcoHIV infection reduces the number of inflammatory monocytes in the brains of infected mice. To evaluate peripheral virus burdens in this experiment, DNA was isolated from spleen cells and quantified by qPCR as described. Buprenorphine did not significantly change the amount of HIV DNA in the periphery of infected mice (**Figure 2D**).

Inflammatory monocytes can harbor HIV and contribute to CNS reservoir reseeding, another important mechanism of HIV neuropathogenesis (25, 29, 41, 68). Thus, inhibiting the migration of specifically HIV infected monocytes into the brain might limit this reseeding and the amount of virus in the brain. To test this, we performed an additional study using the two-week timepoint (**Figure 2E**). Mice were infected with EcoHIV and then given buprenorphine after two weeks of infection. After an additional three weeks, these animals were perfused, brain tissue was harvested, DNA was isolated, and HIV *gag* DNA was quantified as described (Gu; Kim). We found a significant decrease (p<0.05) in the amount of HIV DNA in the brains of buprenorphine treated, Eco HIV infected mice (23.68 +/- 12.12) compared to infected mice (117.2 +/- 72.41) (**Figure 2E**). These data suggest that buprenorphine treatment reduces HIV DNA burdens in the brains of infected mice, potentially by limiting the entry of EcoHIV harboring cells into the CNS, and is not affecting the amount of virus in the periphery that would cause overall decreased viral levels.

### Cognitive impairment is present two weeks after infection and is correlated with increased numbers of inflammatory monocytes in the brain

**Figure 3:**
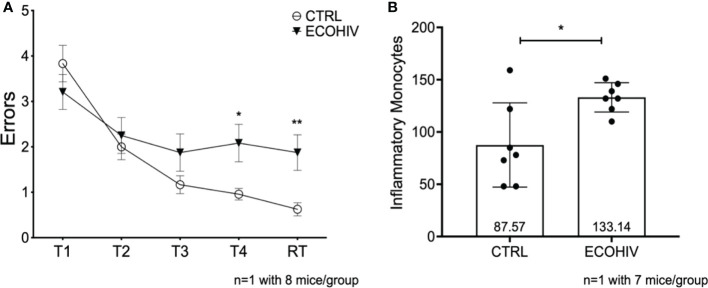
Mice are cognitively impaired after two weeks of infection and have significantly more brain inflammatory monocytes. We tested an early timepoint after infection a time in which cognitive impairment is being established. These data are from one independent experiment with eight mice in each group for water maze testing **(A)** and seven mice in each group for monocyte studies **(B)** Mice were infected with EcoHIV or injected with a diluent control (PBS). After these two weeks, eight mice in each group performed RAWM testing **(A)** EcoHIV infected mice, represented as the closed circles, were cognitively impaired as they made significantly more errors in Trial 4 and the retention trial compared to control mice represented as the open circles (T5 p<0.05, RT p<0.001). These data show that the establishment of EcoHIV-NCI has begun to manifest after two weeks of infection. After which the number of inflammatory monocytes were quantified by flow cytometry as previously described. **(B)**, Data are shown as mean +/- SD. There are significantly more inflammatory monocytes (p<0.05) in the brains of EcoHIV infected mice (133.1 +/- 14.03) compared to control mice (87.57 +/- 40.27) determined by a Student’s t-test. *p<0.05; **p<0.01.

We had not previously examined the NCI status and monocyte entry into the CNS at two weeks after infection, the time point at which we began buprenorphine treatments. Thus, we performed another set of experiments to test these parameters using only EcoHIV infected mice (ECOHIV) and control mice (CTRL). Cognitive impairment was determined in a RAWM test as described above. Our results show that after two weeks of infection, mice were unable to learn and remember where the hidden platform was in Trial 4, T4 (p<0.05), and the retention trial, RT (p<0.01), indicating that they are cognitively impaired (**Figure 3A**). Evaluation of inflammatory monocyte migration to the brain in these mice demonstrated that the animals had significantly elevated numbers of brain inflammatory monocytes at this stage of EcoHIV infection (**Figure 3B**). Monocytes were quantified in immune cells extracted from perfused brains as described above. There was a significant increase in the number of monocytes in the brains of infected mice compared to uninfected control mice (133.13 vs 87.57, p < 0.05 ECOHIV vs CTRL) (**Figure 3B**). These results show that the migration of inflammatory monocytes into the brain may drive the neuropathogenesis we detect at two weeks after infection.

### Buprenorphine reverses EcoHIV-NCI and reduces monocyte migration and viral brain DNA when given four weeks after infection

**Figure 4:**
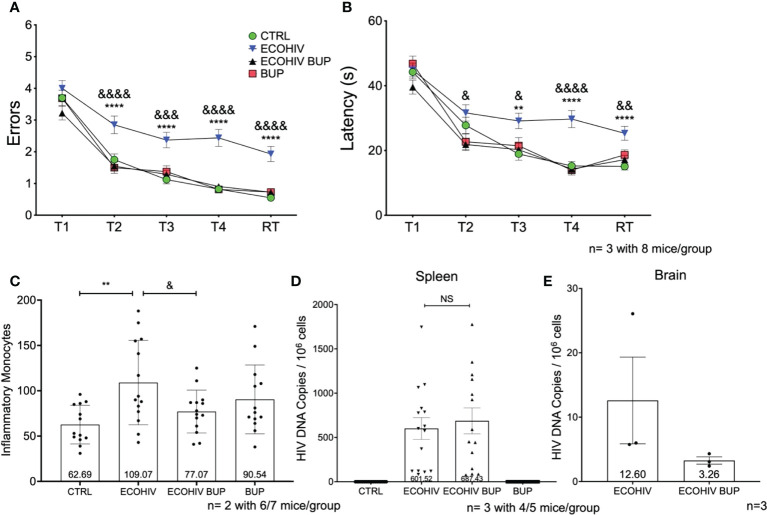
Buprenorphine reverses cognitive impairment and reduces brain inflammatory monocytes when given four weeks post-EcoHIV infection. C57BL/6J mice in each experimental group; CTRL (green circles), ECOHIV (blue inverted triangles), ECOHIV BUP (black triangles), and BUP (red squares) were tested using a radial arm water maze. Mice were infected for four weeks before buprenorphine injections began. After an additional two weeks, mice were assayed for cognitive performance using the RAWM **(A, B)**. EcoHIV infected mice were cognitively impaired after four weeks of infection as these mice made significantly more errors **(A)** and took significantly longer **(B)** to find the hidden platform compared to both CTRL (*) and ECOHIV BUP (&) groups. Data are shown as mean+/- SEM from the last three days of water maze testing. There were a total of three independent experiments, each with eight mice/group for a total of 24 mice in the CTRL, ECOHIV, and ECOHIV BUP groups. Two independent experiments were performed with 8 mice for the BUP group for a total of 16 mice for **(A, B)** Results were analyzed using a one way ANOVA: ECOHIV vs CTRL, **p<0.005, ****p<0.0001. ECOHIV vs ECOHIV BUP, ^&^p<0.05, ^&&^p<0.005, ^&&&^p<0.0005, ^&&&&^p<0.0001. **(C)** Monocytes were quantified as described in the previous experiments and shows the number of brain inflammatory monocytes from a total of 12-14 mice in each group from two independent experiment. The data are shown as mean +/- SD. There is a significant increase (109.07 +/- 46.58) in the number of inflammatory monocytes in the brains of EcoHIV infected mice compared to control (62.69 +/- 21.29, **p<0.01). These data also show that buprenorphine treatment four weeks post infection significantly reduces the number of brain inflammatory monocytes (77.07 +/- 23.68, ^&^p<0.05). **(D)**, These results are also correlated with no change in the spleen HIV DNA between ECOHIV and ECOHIV BUP. **(E)**, DNA was isolated from a section of the right hemisphere from four or five mice in each group. This DNA was then pooled and concentrated. The number of HIV *gag* DNA copies were quantified using ddPCR and normalized to 10^6^ cells. The data are shown as mean +/- SEM. These data are from three independent experiments for CTRL, ECOHIV, and ECOHIV BUP groups, and from two independent experiments for BUP. There is an increase in the number of HIV DNA copies in infected mice (12.60 +/- 6.734) compared to EcoHIV infected mice that were given buprenorphine (3.263 +/- 0.5626). These data suggest that buprenorphine may reduce the amount of virus in the brains of mice when given four weeks after infection. NS, Not Significant.

In the next series of studies, we repeated the experiments shown in **Figure 2** but started buprenorphine treatment four weeks after infection, with cognitive assessment two weeks after buprenorphine treatment and monocyte brain migration quantification, and virological evaluations performed after cognitive testing, three weeks later (**Figures 1**, **4**). The experimental groups were the same as in the two week buprenorphine experiments and the results were remarkably similar. EcoHIV infected mice were cognitively impaired making significantly more errors (T2-RT: p<0.0001) and taking significantly longer (T3: p<0.005, T4-RT: p<0.0001) to find the hidden platform compared to control mice (**Figures 4A, B**, ECOHIV versus CTRL**).** Buprenorphine reversed EcoHIV-NCI (**Figures 4A, B**, ECOHIV BUP vs ECOHIV) as indicated by a significant decrease in the number of errors made (T2, T4, RT: p<0.0001, T3: p<0.0005) and latency (T2, T3: p<0.05, T4: p<0.0001, RT: p<0.005) compared to ECOHIV mice. As in the first experiment series, EcoHIV infected, buprenorphine treated mice had significantly reduced inflammatory monocytes when compared to infected mice (77.07 +/- 23.68, p<0.05 ECOHIV BUP vs ECOHIV). Monocytes from six or seven mice per group were analyzed from two experiments, for a total of 12-14 mice (**Figure 4C**). EcoHIV infected mice had significantly more brain inflammatory monocytes (109.07 +/- 46.58) compared to control mice (62.69 +/- 21.29, p<0.01). Additionally, there were no significant differences among the number of inflammatory monocytes from EcoHIV infected mice that were given buprenorphine, uninfected buprenorphine treated mice, and control mice. At the end of these studies we tested the effects buprenorphine treatment on peripheral HIV DNA (**Figure 4D**). We saw no difference between treated and untreated mice in the amount of HIV DNA in the periphery. Using ddPCR, we also tested the effect of buprenorphine on HIV brain burdens in treated and untreated mice (**Figure 4E**). Consistent with the results shown in **Figure 2E**, buprenorphine, started after four weeks of infection, also decreases the amount of virus in the brains of infected mice.

### Buprenorphine protects neurons from synaptodendritic pruning in the brains of EcoHIV infected mice

**Figure 5:**
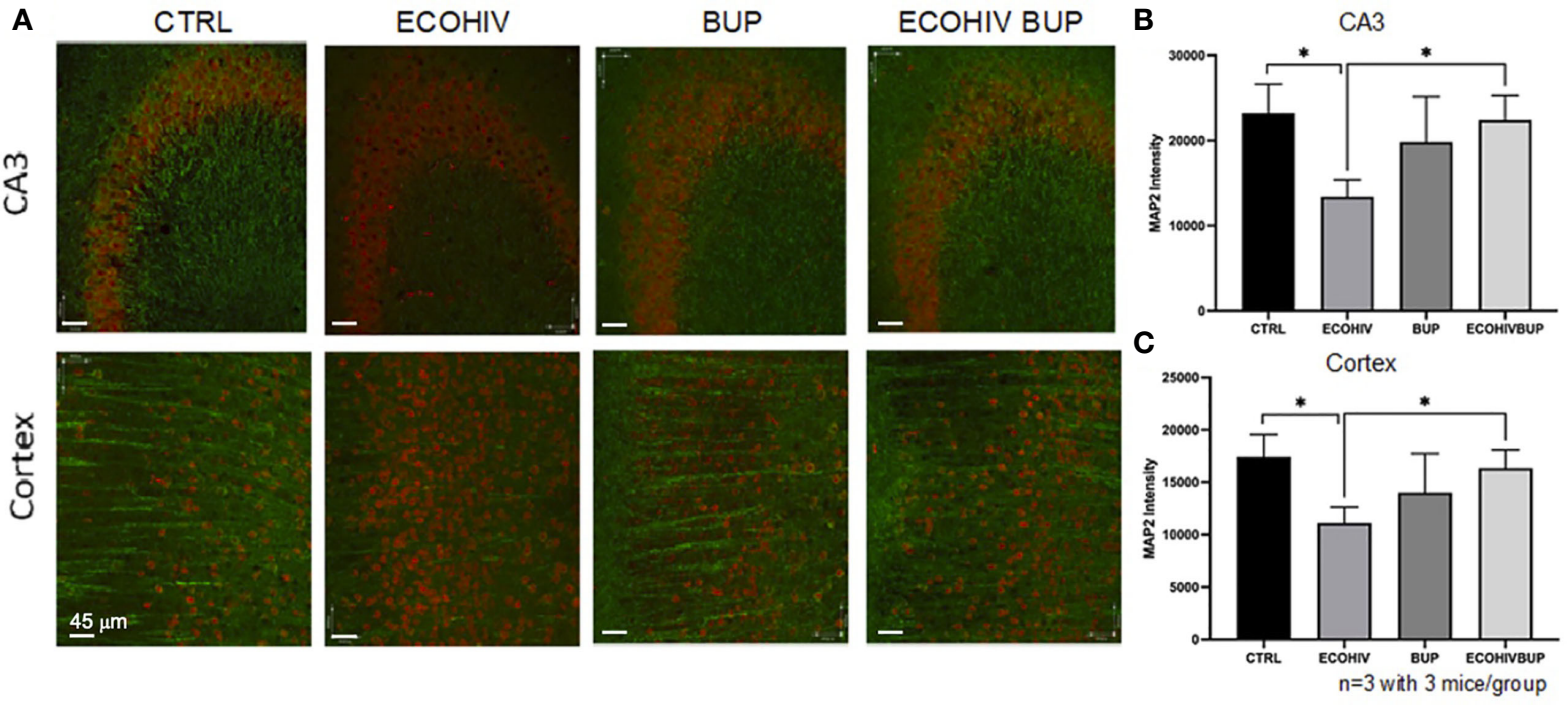
Treatment with buprenorphine protects against EcoHIV mediated synaptodendritic pruning. Brains from three mice in each group per two independent experiments were perfused, frozen, and sectioned for confocal microscopy. The mice used for these experiments were part of the approach in which buprenorphine was administered two weeks post infection. The cortex and CA3 region of the hippocampus of the brain were stained for NeuN and MAP2 to identify the neuronal cell body and dendritic processes. **(A)** shows representative images from these two regions from one mouse in each experimental group. The red staining defines the neuronal cell body (NeuN) and the green staining defines the dendritic processes (MAP2). There is a significant loss of MAP2 intensity from the CA3 region **(B)** and the cortex **(C)** of EcoHIV infected mice compared to control and compared to EcoHIV infected, buprenorphine treated mice. These results show that buprenorphine protects against EcoHIV mediated synaptodendritic pruning.

Synaptodendritic injury is a pathological hallmark of HIV-NCI in PWH on suppressive ART, and this hallmark is reproduced in infected mice with EcoHIV-NCI (13, 69). This injury, in both human and murine NCI, occurs in the absence of significant neuronal death (13, 69). Thus, it can be prevented or reversed upon reduction of the pathogenic HIV stimulus in the brain, leading to mitigation of cognitive dysfunction (13). Here we tested whether reversal of EcoHIV-NCI by buprenorphine correlates with improvement in synaptodendritic integrity by examining MAP2 intensity by immunofluorescent staining and confocal microscopy (**Figure 5**). Brains were isolated from two mice from each group in two independent experiments. Ten to 20 30um sections were stained with MAP2 and NeuN to label the dendritic process and neuronal cell body, respectively, as described in the methods. Ten to 20 images of the CA3 region of the hippocampus and cortex regions of the brains were taken in each brain section from each mouse per group from three independent experiments. As we showed previously (13), EcoHIV infection results in synaptodendritic pruning (**Figures 5A-C**) as indicated by the decrease in MAP2 intensity in the CA3 region of the hippocampus and cortex compared to control (p<0.05). Buprenorphine therapy reversed the EcoHIV mediated neuronal injury as there was a significant increase in MAP2 intensity in the CA3 region and cortex of ECOHIV BUP mice compared to ECOHIV mice (p<0.05) (**Figures 5A–C**).

## Discussion

Despite effective ART that inhibits viral replication and greatly increases the lifespan of PWH, HIV-NCI still persists in 15-50% of PWH. HIV-NCI negatively impacts the quality of life and is an independent risk factor for mortality (70). OUD is believed to be associated with worse neurocognitive impairment in PWH and can result in ART interruption that can contribute further to NCI (71–73). The impact of opioids on the mechanisms that result from HIV brain disease in individuals on suppressive ART has not been fully defined. This limited understanding of mechanisms that mediate mild HIV-NCI pathogenesis and its potential exacerbation with opioid use, despite ART, has resulted in a lack of therapeutic strategies for the treatment of NCI in the absence or presence of OUD. Buprenorphine is an OAT that treats OUD (51, 52, 74). It has an improved safety profile compared to another OAT, methadone, due to its unique properties as a partial agonist for MOR and full antagonist for KOR (52). In some studies, people with OUD taking buprenorphine had improved cognitive outcomes compared to those who did not (55, 75). Data in another study also suggest that buprenorphine improves cognitive outcomes in people with OUD compared to their own baseline before buprenorphine treatment (56). The mechanisms by which buprenorphine improves cognition in PWH with or without OUD still need to be characterized. This is a significant gap in our ability to treat HIV CNS disease. We propose that buprenorphine, in addition to its being an OAT, can be an effective interventional therapy for HIV-NCI in PWH regardless of OUD.

To test the ability of buprenorphine to be a therapy for HIV-NCI we used the EcoHIV mouse model. This model has many advantages for the study of HIV infection and potential therapies. These include the expression of all HIV genes except gp120. EcoHIV preserves HIV cellular tropism to CD4+ T cells, macrophages, and microglia, and not to other cell types. Infection with EcoHIV also induces immune responses to HIV proteins that enable vaccine studies. Importantly for our studies, infection with this virus results in neurocognitive disease impairing learning, memory, and fear responses in all infected mice, a disease highly similar to mild chronic HIV neurocognitive impairment in PWH on virus-suppressive ART as well as other HIV-associated chronic abnormalities typically seen in PWH on ART, including gut, lung, and brain microvascular diseases. EcoHIV infection of the brain also reflects the low level HIV brain burdens in humans.

The results shown in **Figures 2**–**4** indicate that the continuous migration of inflammatory monocytes into the brain at three, five, and seven weeks of infection contributes to the development and persistence of cognitive impairment. The increased entry of inflammatory monocytes is an important mediator of HIV neuropathogenesis. Several studies in mice and non-human primates showed the ability of monocytes to harbor the virus and enter the brain. This was associated with neuronal damage (11, 27, 76, 77). It has also been shown that HIV DNA in peripheral blood monocytes correlated with cognitive impairment in PWH (27, 28, 45). Thus, we propose that this migration is reduced by daily buprenorphine treatments started at two or four weeks after infection. Buprenorphine treatment started at those timepoints also decreased the amount of HIV DNA in the brain. One study in non-human primates showed treatment with natalizumab reduced the number of SIV RNA positive cells in the brain and this correlated with decreased neuronal damage (78). This suggests that limiting the entry of virus harboring cells will reduce neuronal damage and cognitive function. Thus, we hypothesize that limiting the entry of inflammatory monocytes into the brain, and specifically those harboring HIV, decreases CNS viral reservoir reseeding. As such, buprenorphine could mitigate EcoHIV mediated CNS damage, and treat and reverse EcoHIV-NCI, in part by its actions on inflammatory monocytes.

These are important findings as there are currently no approved therapies for HIV-NCI for PWH regardless of successful ART, despite several potential candidates. HIV-NCI interventions have been tested in animal models of HIV neuropathogenesis, including in EcoHIV infected mice. These are treatment with minocycline, memantine, intranasal insulin, 6-diazo-5-oxo-L-norleucine (DON), the DON prodrug, JHU083 and polyinosinicpolycytidylic acid (poly I:C) (12, 14–17). All of these target important processes in the development of HIV-NCI including brain glucose metabolism (13), brain glutamate metabolism (17), or brain exosome production (79). However, to our knowledge, buprenorphine is the first therapy that targets the migration of inflammatory monocytes into the CNS, a key process required for the establishment and maintenance of HIV-NCI. By reducing this migration, buprenorphine can reverse EcoHIV-NCI. Buprenorphine was originally developed as an analgesic and not an OAT (80, 81). It is often prescribed to treat pain related to HIV-associated peripheral neuropathy, another complication associated with HIV infection (81). Thus, it is already used clinically for conditions other than OUD. Additionally, due to its unique pharmacology and the presence of a ceiling effect, buprenorphine has a lower risk of addiction and overdose compared to other opioids such as morphine.

It is important to identify the mechanisms by which buprenorphine reverses cognitive impairment and reduces monocyte migration. One potential mechanism by which buprenorphine reduces the ability of mature monocytes to cross the BBB is through receptor desensitization. Chemokine receptors, such as CCR2 the receptor for CCL2, as well as the opioid receptors MOR and KOR, belong to a receptor family known as G protein coupled receptors, GPCR (82, 83). Upon activation, these receptors can form heterodimers which modulate their receptor activity (84–86). We propose that concomitant treatment of buprenorphine to an inflammatory environment in which CCL2 is elevated, may cause the formation of MOR/CCR2 and/or KOR/CCR2 heterodimers, thus diminishing the migratory response of these cells. These studies are ongoing. While we show that buprenorphine limits the entry of inflammatory monocytes into the brains of EcoHIV infected mice, additional mechanisms may also contribute to the reversal of EcoHIV-NCI. Buprenorphine can impact brain resident cells such as microglia, macrophages, astrocytes, and neurons. Further characterization of the mechanisms by which buprenorphine reverses EcoHIV-NCI and affects additional cell types will enable the development of next-generation therapies for treating HIV-NCI. Thus, buprenorphine may be a promising candidate for the treatment of HIV-NCI regardless of OUD, as well as for other inflammatory brain pathologies for which there are no therapies.

These findings are highly significant, as HIV-NCI is a prevalent comorbidity of HIV infection. As such, it is imperative to develop strategies to mitigate and eliminate this condition. Buprenorphine reduces many mechanisms that mediate HIV neuropathogenesis including inflammatory monocyte migration, neuronal damage, and possibly virus within the CNS. Our impactful findings show that buprenorphine reverses EcoHIV-NCI in part by its effects on these mechanisms. Thus, buprenorphine may be a promising candidate for the treatment of HIV-NCI regardless of OUD as well as other inflammatory brain pathologies for which there are no therapies.

## Data availability statement

The original contributions presented in the study are included in the article/**Supplementary Material**. Further inquiries can be directed to the corresponding author.

## Ethics statement

The animal study was reviewed and approved by Icahn School of Medicine at Mount Sinai and the Albert Einstein College of Medicine Institutional Animal Care and Use Committees.

## Author contributions

AM, JK, HH, B-HK, and WC performed experiments and contributed to data acquisition and analysis. AM, JK, JB, and DV contributed to the conceptualization, funding, and resources for the studies. AM, JK, JB, and DV developed, wrote, and reviewed the manuscript. All authors contributed to the article and approved the submitted version.

## Funding

These studies were supported by National Institute of Health grants R01DA041931 (JB, JK, AM, HH, WC, and DV), R01DA048609 (JB and AM), R01DA044584 (JB), R01MH112391 (JB), U01DA053629 (DV and JK), R56NS119439 (B-HK, DV, and JK), R01DA052844 (DV and JK), 1RF1NS119438 (JK, B-HK, and DV), as well as the Burroughs Wellcome Fund award: 1TL1TR002557 (AM).

## Acknowledgments

We would like to thank all members of the Berman and Volsky laboratories for their support. We would especially like to thank Dr. Eran Hadas and Dr. Samuel Martinez-Meza for their help and technical expertise.

## Conflict of interest

The authors declare that the research was conducted in the absence of any commercial or financial relationships that could be construed as a potential conflict of interest.

## Publisher’s note

All claims expressed in this article are solely those of the authors and do not necessarily represent those of their affiliated organizations, or those of the publisher, the editors and the reviewers. Any product that may be evaluated in this article, or claim that may be made by its manufacturer, is not guaranteed or endorsed by the publisher.

## Supplementary material

Supplementary Figure 1 Inflammatory brain monocyte gating strategy. This figure represents the gating strategy used to identify brain inflammatory monocytes. These flow cytometry plots are representative images from an EcoHIV infected mouse used in our studies. The first panel represents all cells obtained from cell suspension and percoll gradient density centrifugation. Singlets are gated and shown in panel 2. CD11b is used to identify the immune cell population, panel 3, and contains the cells that enter the brain from the periphery as well as the resident immune cells. In panel 4, we use Ly6G to identify and exclude the neutrophils that are shown in the black rectangle. The analysis proceeds with the Ly6G negative population shown in the red rectangle. In Panel 5, the cells are stratified by CD45 expression. The black rectangle contains the CD45 intermediate cells, including microglia and macrophages. In the red rectangle are the CD45 hi cells that contain the inflammatory monocytes. The last part of the analysis, panel 5, identifies the Ly6C+ cells from the previous red gate (panel 5) that are the inflammatory brain monocytes.
