# Supplementary figures and images for: Buprenorphine reverses neurocognitive impairment in EcoHIV infected mice: A potential therapy for HIV-NCI
Source: Front Immunol. 2022 Oct 7;13:1004985. doi: 10.3389/fimmu.2022.1004985 (PMC9585248; doi:10.3389/fimmu.2022.1004985)

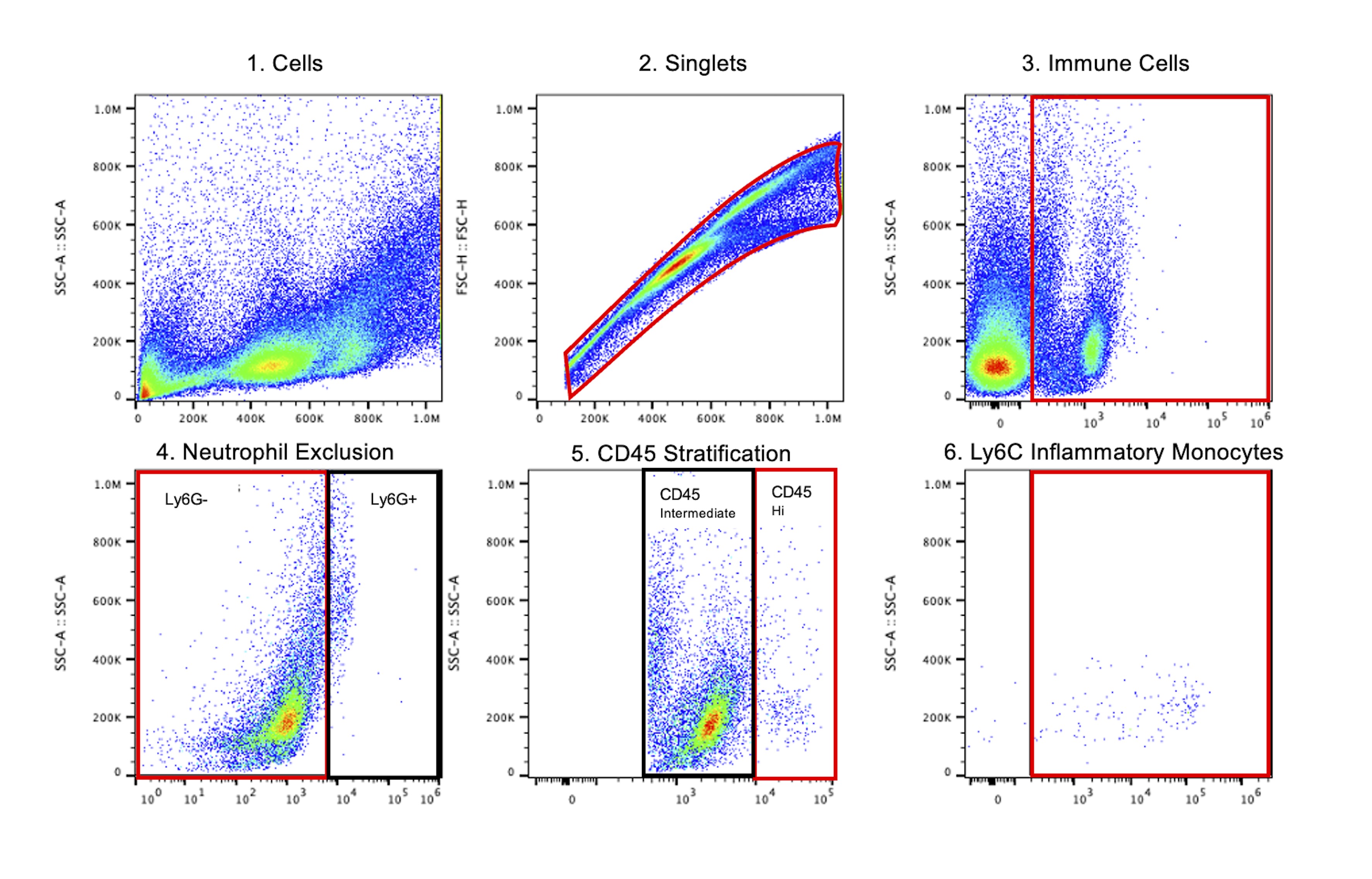

Supplement: Supplementary Figure 1 — Inflammatory brain monocyte gating strategy. This figure represents the gating strategy used to identify brain inflammatory monocytes. These flow cytometry plots are representative images from an EcoHIV infected mouse used in our studies. The first panel represents all cells obtained from cell suspension and percoll gradient density centrifugation. Singlets are gated and shown in panel 2. CD11b is used to identify the immune cell population, panel 3, and contains the cells that enter the brain from the periphery as well as the resident immune cells. In panel 4, we use Ly6G to identify and exclude the neutrophils that are shown in the black rectangle. The analysis proceeds with the Ly6G negative population shown in the red rectangle. In Panel 5, the cells are stratified by CD45 expression. The black rectangle contains the CD45 intermediate cells, including microglia and macrophages. In the red rectangle are the CD45 hi cells that contain the inflammatory monocytes. The last part of the analysis, panel 5, identifies the Ly6C+ cells from the previous red gate (panel 5) that are the inflammatory brain monocytes. [file Image_1.jpeg]
